# Radiation-induced cavernous malformation after stereotactic radiosurgery for cavernous sinus meningioma: a case report

**DOI:** 10.1186/s12883-020-01995-8

**Published:** 2020-11-20

**Authors:** Zuan Yu, Bin Huang, Risheng Liang

**Affiliations:** Department of Neurosurgery, Fujian Medical University Union Hospital, No. 29 Xinquan Road, Gulou District, Fuzhou City, Fujian Province China

**Keywords:** CM: cavernous malformation, CS: cavernous sinus, CSM: cavernous sinus meningioma, Complication, RICM: radiation-induced cavernous malformation, SRS: stereotactic radiosurgery

## Abstract

**Background:**

Radiation-induced cavernous malformation (RICM) is a rare sequela of stereotactic radiosurgery (SRS) treatment of intracranial tumors. To date, no study reported on RICM after SRS for meningiomas originating from the skull base. The relationship between locus of initial meningioma and RICM has not been studied.

**Case presentation:**

A 57-year-old woman presented with persistent headaches and blepharoptosis at initial episode. MRI disclosed a right parasellar lesion, diagnosed as a cavernous sinus meningioma (CSM). After receiving a single-fractionated SRS, headache relieved, but blepharoptosis did not significantly improve. Three years and three months later, she returned with headaches and dizziness. MRI showed an enlarged CSM. Moreover, a new mass-like lesion, suspected hemangioma, appeared in the nearby right temporal lobe. After surgical removal of the new lesion and the CSM, the patient’s neurological symptoms significantly improved. Pathology confirmed CSM and temporal RICM.

**Conclusions:**

We report the first rare case of RICM occurring after SRS for CSM. The RICM may be in the same region as the initial tumor. Surgical intervention was preferred for symptomatic RICM and initial meningioma. We recommend long-term regular followup MRIs for patients with meningioma after SRS treatment.

## Background

Due to the proximity of CSMs to critical cranial nerve (CN) and vascular structures, complete resection using an endoscopic or transcranial skull base approach comes with high surgical risks. SRS is an effective treatment for CSMs after their resection or as an upfront treatment [1–3]. While many studies have reported on the advantages and disadvantages of SRS as the primary or adjuvant management for CSMs [2–10], few have highlighted the scarce complications of this treatment.

Radiation-induced cavernous malformation (RICM) is a rare sequela after radiotherapy for intracranial tumors. Increased initial intracranial tumors including glioma, ependymoma, medulloblastoma, and cavernoma [11, 12], have been reported with few studies demonstrating the formation of RICM after SRS for meningioma. Miyamoto T, et al. were first to reported a case of suspected cavernous malformation (CM) in 1994 [13]. The first radiologic and pathologic confirmation for RICM induced by meningioma radiotherapy was reported in 2014 [11]. Of note, the location of RICM’s initial meningioma after radiotherapy has not yet been reported either in the cavernous sinus (CS) or in the whole skull base.

## Case presentation

### History of presenting illness

**Fig. 1:**
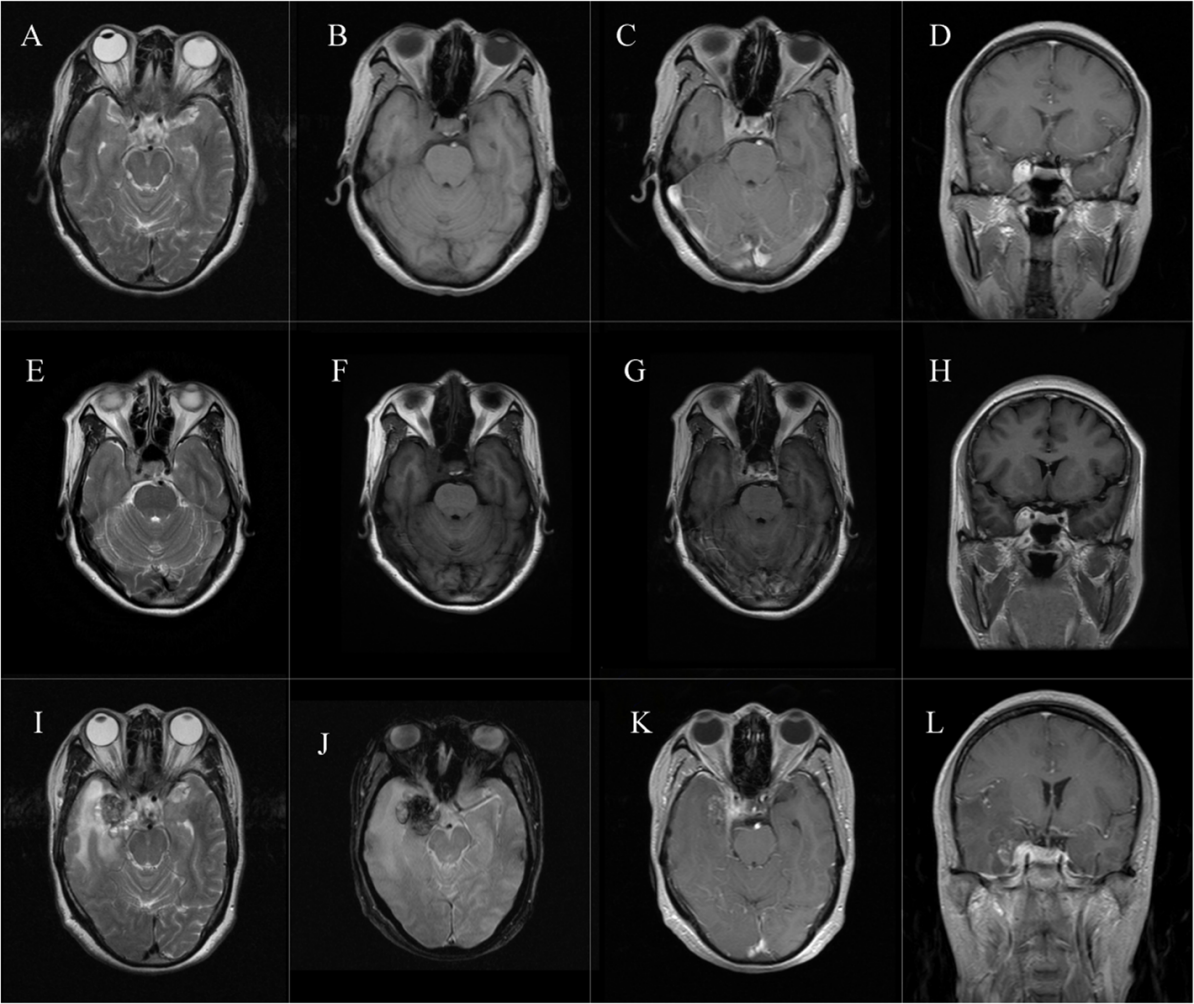
MR images before surgery: MR images before SRS show irregular nidus with abnormal signal in the right parasellar region(**a**, **b**, **c**, **d**).)MR images 8 months after SRS show size of CSM is similar to that before treatment (**e**, **f**, **g**, **h**). MR images after admission show that the size of CSM was larger than that of 8 months after SRS. Another lesion appears on the right temporal lobe (**i**, **j**, **k**, **l**)

We report a case of RICM after SRS treatment for a CSM. A 57-year-old female presented with a persistent headache and right blepharoptosis in December 2015. The headache was characterized as a persistent needle-like pain in the right orbital and cervico-occipital region. Other symptoms included visual ghosting and tearing. Cranial MRI revealed an abnormal signal in the right parasellar region approximately 1.4 × 1.9 × 1.9 cm in size with an unclear boundary. It was slightly hypointense on T1-weighted images (T1WI) and slightly hyperintense on T2-weighted images (T2WI). The MRI signal enhanced significantly after administration of contrast (Fig. 1). The lesion was diagnosed as a right CSM, invading the right CS and circumvoluting the right internal carotid artery.

Due to the location of the nidus, craniotomy risks, and total resection feasibility, the patient received single-fractionated SRS with a central dose of 24 Gy and a margin dose of 12 Gy. Fifteen days following treatment, the patient’s headaches were gone; however, there was no significant improvement in right eyelid droop or double vision. Despite resolution of the headache, the ptosis persisted 8 months after treatment, and MRI reexamination showed that the size of the CSM was similar to that before SRS (Fig. 1). In that time, the patient underwent irregular followup.

Three years and three months subsequent to the initial diagnosis, she returned to the hospital due to worsening right eyelid droop and headaches accompanied by dizziness. MRI showed that the original right CS lesion had grown to approximately 2.9 × 2.2 × 1.9 cm with an unclear boundary. It appeared slightly hypointense on T1WI images, slightly hyperintense on T2WI images, and progressively and homogeneously enhanced on enhancive images. Concurrently, a new lesion had appeared nearby in the right temporal lobe and was approximately 2.85 × 2.65 × 2.0 cm on T2-star weighted images. MRI images of the new lesion were slightly hypointense with an enormous area of edema in the temporal lobe around the nidus on T2WI images, hypointense on T2-star weighted images, and iso- or hypointense on T1WI images. After contrast was injected, irregularly patchy enhancement within the lesion and strip enhancement around the lesion appeared on T1WI. The radiological appearance of the new nidus was consistent with hemangioma from the old bleed (Fig. 1).

### Diagnosis and treatment

**Fig. 2:**
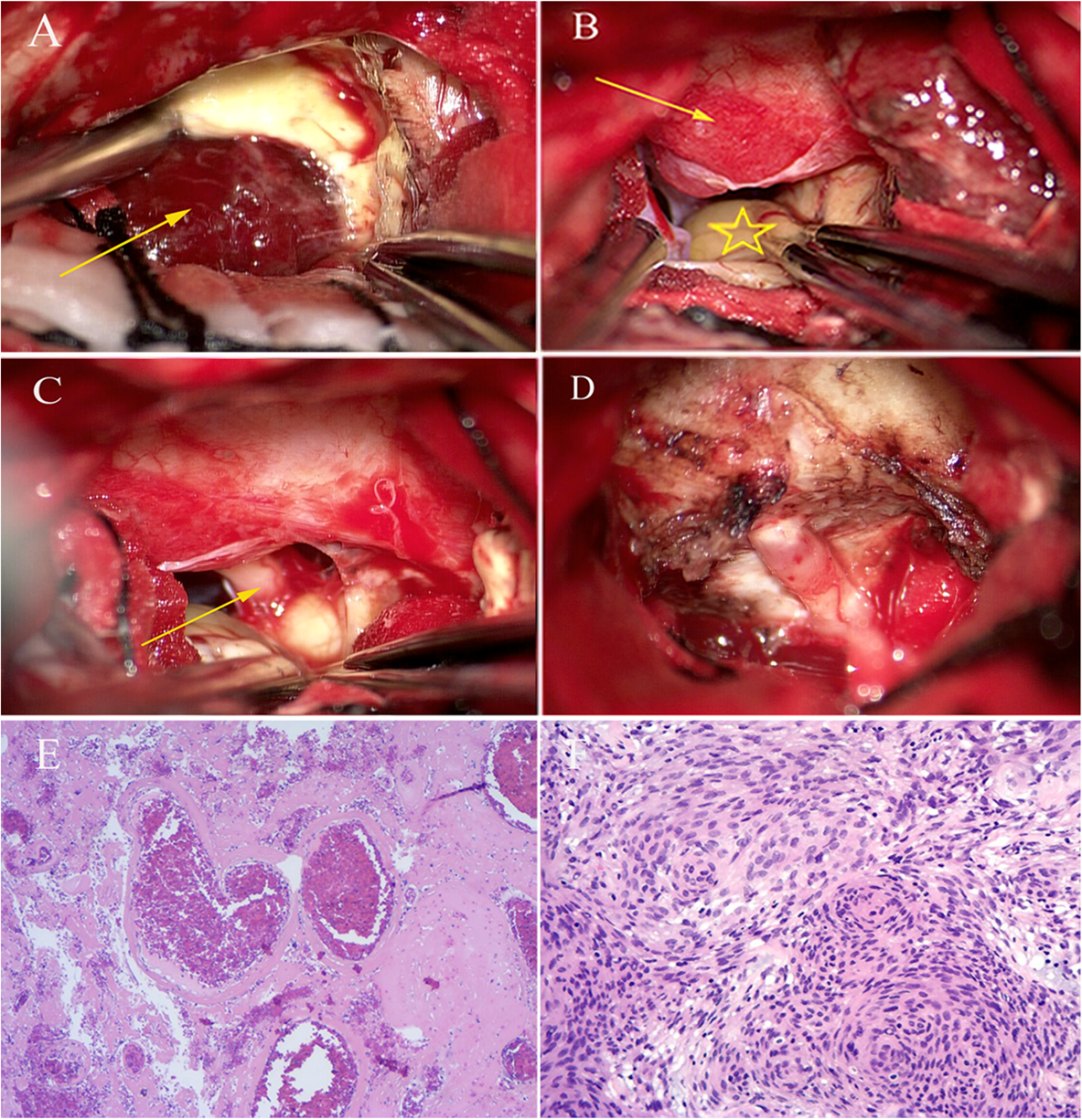
Intraoperative images of removal tumors and photomicrographs of the surgical specimens. **a**. Yellowing white matter and RICM in the anterior temporal lobe (arrow). **b**. Lesion on the surface of CS (arrow), infratentorial herniation of the uncus of the temporal lobe (star). Oculomotor nerve is not visible. **c**. Inferiorly compressed distal segment of the cisternal oculomotor nerve (arrow), tumor encasement of the oculomotor nerve around its entrance into the CS. A thin layer of tumor tissue can be observed on the tentorial margin. **d**. Structures can be observed after tumor removal. **e**. Tumor cells of CSM are lobularly arranged and partially spiral-like, the tumor nucleus is elliptical, the nucleolus is not obvious, and the cytoplasm is unclear. **f**. Microscopically, there were old bleeding and malformed vessels in the nidus of the right temporal lobe, and dilated vessels were filled with red blood cells and lacked brain tissue. Cells were positive for CD31 and CD34 immunohistochemically

Due to the clinical manifestations and results of the pre-and post-admission examinations, we assumed this time that the symptoms were caused by the new nidus in the right temporal lobe causing edema of the surrounding brain tissue and an increase in the intracranial pressure, leading to headache and dizziness. The patient decided to undergo surgical treatment after being informed of the possible risks of worsening headache, hemangioma re-bleed, progression of the right temporal lobe edema, and difficulty in controlling the edema with drug-only treatment. With informed consent from the patient and her family, surgery was performed under general anesthesia in order to remove the space-occupying lesion in the right temporal lobe and the CSM using the traditional pterional approach. During surgery, the lesion in the medial temporal lobe appeared purplish-red with a slightly tough texture, had a relatively complete envelope, and was well-defined with a size of 3.0 × 2.8 × 2.5 cm. After severing the small feeding arteries, we completely resected the lesion (Fig. 2a). We also removed the tumor tissue on the lateral wall and the upper surface of the CS, which had a medium texture and bled easily (Fig. 2a, b). The tumor tissue inside the CS was not resected (Fig. 2d).

### Postsurgical course

**Fig. 3:**
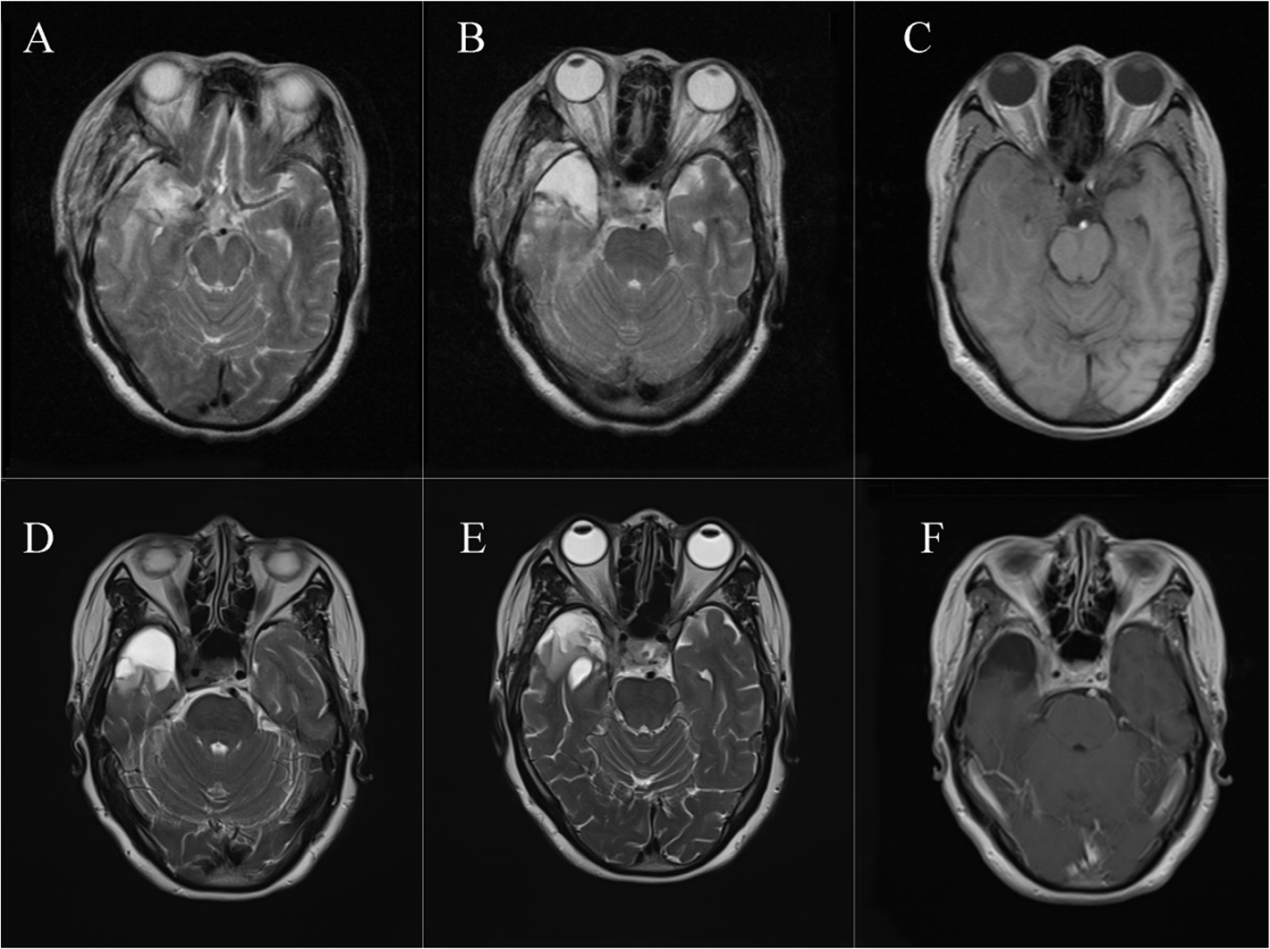
MR images after surgery: MR images 15 days after surgery (**a**, **b**, **c**). MR images 6 months after surgery (**d**, **e**, **f**). The new lesion was completely removed, and the CSM achieved partial removal. The temporal lobe edema area has decreased remarkably on the T2WI images

The patient suffered from short-term upward movement disorder of the right eye after the operation, which may have been a result of right oculomotor nerve paresis caused by intraoperative retraction. The symptoms that included headaches, dizziness, right eyelid droop, and right eyeball movement disorder improved after medication treatment and rehabilitation exercises. The pathological diagnosis of the parasellar lesion was endothelial meningioma (WHO grade I), and immunohistochemical staining showed the tumor cells to be positive for EMA, vimentin, with 2% positive for Ki-67 indices, and S100 and GFAP negative (Fig. 2d). The nidus of the right temporal lobe was pathologically diagnosed as a CM with a hemorrhage, and cells were immunohistochemically positive for CD31 and CD34 (Fig. 2f). MR images 15 days after surgery revealed that the RICM was completely removed and that the CSM had achieved partial removal (Fig. 3a, b, c). One month after the operation, ptosis of the right eyelid disappeared, and movements of the right eyeball were normal. Six months after the operation, the right temporal lobe edema had almost subsided on the re-examination images, and both the headaches and blepharoptosis had completely resolved (Fig. 3d, e, f).

## Discussion and conclusions

With a good long term tumor control rate and low morbidity, SRS offers a viable alternative for treating skull base meningioma [8, 9]. Review of relevant literature brought out some interesting studies. Minniti, et al. compiled 18 studies with a total of 2919 skull base meningiomas treated with gamma knife radiosurgery (GKRS) [14]. The five-year control rate was 91%. Seven of these studies (1626 skull base meningiomas) reported a 10-year averaged control rate of 87.6%. Complications after SRS, including new or aggravated CN deficit and hydrocephalus, although relatively uncommon, should be expectantly monitored [2, 3, 10]. From the records of 200 patients with CSM who had undergone GKRS, 25 patients (12.5%) exhibited delayed onset of additional CN symptoms [10]. The North American Gamma Knife Consortium reported an unfavorable outcome occurring in 149 (20.4%) of the 769 patients [2]. The occurrence of complications may be due to the location, size, and nature of the primary tumor. For example, a delayed endocrinopathy may occur in parasellar and sellar meningioma after GKRS [2, 15–17]. It is recommended that the thyroid should be periodically evaluated in these patients [2]. Patients who had CSM treated with GKRS may even develop a rare carotid artery occlusion [18, 19].

RICM is also a radiotherapy complication for intracranial tumors, especially meningiomas. The pathophysiological mechanisms for RICM’s development remain unclear. Two hypotheses have been proposed [20]. First, the cavernous malformations may be present before radiation, though radiographically occult, and radiation induces their growth. Second, radiation therapy may induce the cavernous hemangioma, resulting in a vessel wall necrosis and changes that include cell swelling, dilation of the vessel lumen, hyalinization, fibrosis, and mineralization that predisposes to CM formation [12, 21], further vessel changes after radiation may be dose-dependent [22, 23]. Genetic mutations may also play a role in the development of cavernous hemangiomas [20, 24].

RICM is quite rare. According to our literature review from 1994 to 2018, only six cases of meningiomas have been diagnosed with RICM after conventional radiotherapy or SRS (Table 1). Of these six cases, only two were radiologically and pathologically confirmed, while the remaining four cases were diagnosed only via MRI. Based on the classification scheme of Zabramski (Table 2) [26], RICMs reported by Nagy G were type II lesions at presentation. In our case, MRI of the RICM was consistent with type III characteristics, which was later pathologically confirmed.

**Table 1:** Summary of published cases of RICM after initial Meningioma Radiotherapy

Patient	Author/year	Sex	Age(Y)	Presentation when diagnosis of RICM	Location of initial meningioma	Location of RICM	Total radiation dose (Gy)	Time from radiation to diagnosis of RICM (yr)	Radiotherapy	Surgery for RICM	Pathological diagnosis of RICM
1	Miyamoto et al.1994 [13]	F	41	Headache, homonymous hemianopia	Right parietotemporal	Right thalamus and left caudate nucleus	50	3	Conventional	N	N
2	Ruggeri AG et al.2014 [11]	F	46	Headache	Left Frontal lobe	Bilateral Frontal lobe	60	3	Conventional	Y	Y
3	Nagy G et al.2018 [25]	Unkown	75	Asymptomatic	Unkown	Temporal lobe	26	2	SRS	N	N
4	Nagy G et al.2018 [25]	Unkown	45	ICH	Unkown	Pons	30	10	SRS	N	N
5	Nagy G et al.2018 [25]	Unkown	47	Asymptomatic	Unkown	Tmporal lobe	36	21	SRS	N	N
6	Nagy G et al.2018 [25]	Unkown	46	Seizure, cognitive decline, visual loss	Unkown	Occipitai lobe	Unkown	7	SRS	Y	Y

*ICH* intracranial hemorrhage, *RICM* radiation-induced cavernous malformation, *SRS* stereotactic radiosurgery

**Table 2:** MRI Classification of Cavernous Malformations

Lesion type	MRI characteristics
I	T1: hyperintense core, T2: hyper- or hypointense core with surrounding hypointense rims
II	T1: reticulated mixed signal core T2: reticulated mixed signal core with surrounding hypointense rim
III	T1: iso- or hypointense T2: hypointense with a hypointense rim that magnifies the size of the lesion GE: hypointense with greater magnification than T2 images
IV	T1: poorly seen or not visualized at all T2: poorly seen or not visualized at all GE: punctate hypointense lesions

*GE* gradient-echo, *MRI* magnetic resonance imaging

Adapted from the classification of Zabramski et al. [26]

According to the literature review, the locations wherein RICMs developed after conventional whole-brain radiotherapy may be random, either in the same area as the initial meningioma or in a different area, with possibly multiple nidi simultaneously emerging. While RICM after SRS treatment may be single, the location of the initial meningiomas was not specified in the literature (Table 1). None of these 6 RICMs cases was reported with the initial meningioma located in the skull base. The CSM in our case was located in the same region as the RICM of the right temporal lobe that developed after SRS treatment. We propose that the relationship between the location of initial meningioma and RICM may be related to how radiotherapy was conducted. We believe that RICM formation after SRS is more likely to be in the same region as the initial meningioma due to accurate localization and precise transmission of radiation. However, more cases are required to prove whether the positional relationship between the primary meningioma and secondary RICM is related to the radiotherapy method.

Symptoms disappeared after surgical removal of two RICMs in the patient with drug-resistant headache [11]. In Nagy’s series, a patient whose RICM was located in the occipital lobe was observed with pathological manifestation of epilepsy, functional cognitive decline, and blurred vision. These neurological conditions improved postoperatively, and the patient was able to discontinue corticosteroids and anticonvulsant medication [26]. In our case, repeated chronic hemorrhage caused by RICM resulted in exacerbated CN deficits and a decline in the quality of life. The neurological condition improved after surgical treatment of RICM and CSM. Therefore, surgical intervention may be a better choice for RICM cases with obvious symptoms. Moreover, for patients after SRS treatment, the initial meningioma can be simultaneously treated. Asymptomatic RICM cases may be selectively observed, but attention should be paid to the possibility of chronic bleeding and enlargement of the nidus.

The median detection time of RICM after cranial irradiation is 8 to 12 years [27–29], and the length of this interval may be dose-dependent [22, 23]. The time interval for RICM for meningioma has been reported to be as short as 2 years from the time of the radiation therapy to the diagnosis, with a maximum of 21 years [25]. Therefore, long term or even lifelong regular MRI follow-up examination is necessary for meningioma patients treated with SRS.

In conclusion, we report the first rare case of RICM occurring after SRS for CSM. The RICM may be in the same region as the initial tumor. Surgical intervention was preferred for symptomatic RICM and initial meningioma. We recommend long-term regular followup MRIs for patients with meningioma after SRS treatment.

## Data Availability

All data generated during the project will be made freely available upon reasonable request. There are no security, licensing, or ethical issues related to these data.

## Abbreviations

CM: Cavernous malformation
CN: Cranial nerve
CS: Cavernous sinus
CSM: Cavernous sinus meningioma
MRI: Magnetic resonance imaging
RICM: Radiation-induced cavernous malformation
SRS: Stereotactic radiosurgery
WHO: World health organization
